# Supplementary material for: Alkalinity responses to climate warming destabilise the Earth’s thermostat
Source: Nat Commun. 2023 Mar 24;14:1648. doi: 10.1038/s41467-023-37165-w (PMC10039064; doi:10.1038/s41467-023-37165-w)
Supplement: Supplementary file 1 — Supplementary Information [file 41467_2023_37165_MOESM1_ESM.pdf]

Supplementary Information

to:

Alkalinity responses to climate warming destabilise the Earth's thermostat

Authors: Nele Lehmann[1,2,3*], Tobias Stacke[1,4], Sebastian Lehmann[5], Hugues Lantuit[2,6], John Gosse[7], Chantal Mears[1], Jens Hartmann[8], Helmuth Thomas[1,3*]

[1]Institute of Carbon Cycles, Helmholtz-Zentrum Hereon; Geesthacht, Germany

[2]Alfred Wegener Institute Helmholtz Centre for Polar and Marine Research; Potsdam, Germany

[3]Institute for Chemistry and Biology of the Marine Environment (ICBM), University of Oldenburg, Oldenburg, Germany

[4]now at Max Planck Institute for Meteorology; Hamburg, Germany

[5]Private investigator; Buchholz in der Nordheide, Germany

[6]Institute of Geosciences, University of Potsdam; Potsdam, Germany

[7]Department of Earth and Environmental Sciences, Dalhousie University; Halifax, NS, Canada

[8]Institute for Geology, Center for Earth System Research and Sustainability (CEN), University Hamburg; Hamburg, Germany

*Corresponding authors. Emails: nele.lehmann@hereon.de and helmuth.thomas@hereon.de

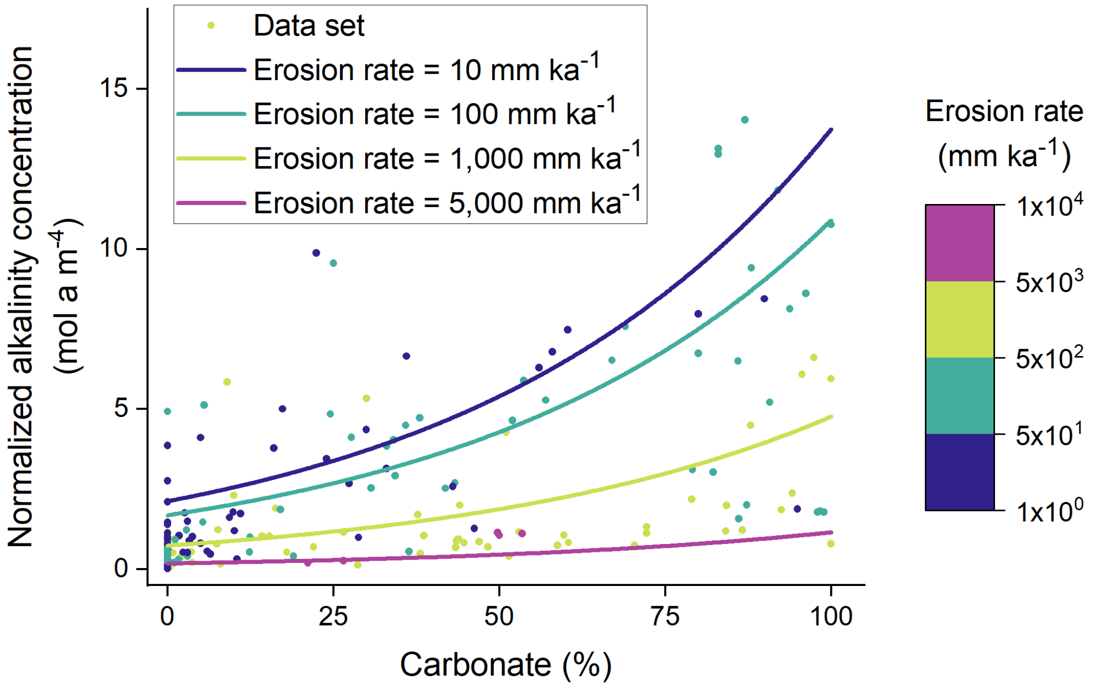

Supplementary Fig. 1: Normalized alkalinity concentration in relation to areal carbonate proportion. Dots are observations. The fitted lines show the model outputs of our final model M5 for different erosion rates. The other covariates are kept constant (MAT = 10°C, catchment area = 1000 km^2, soil regolith thickness = 15 m).

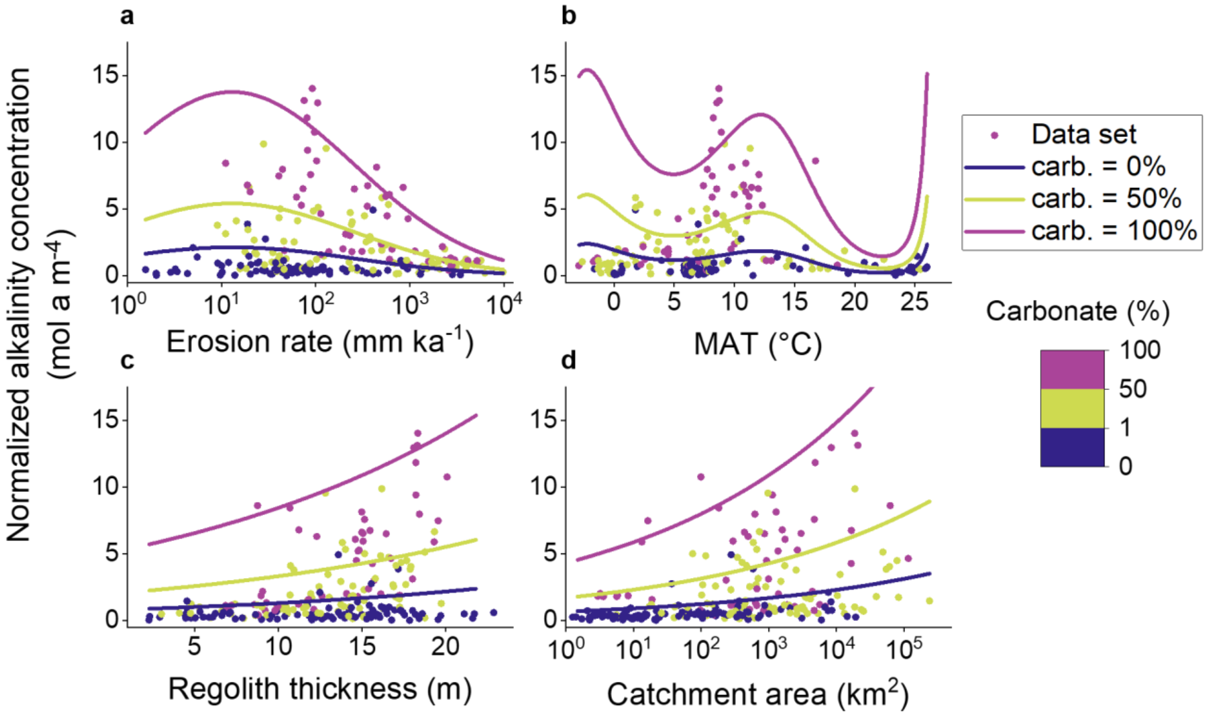

Supplementary Fig. 2: Normalized alkalinity concentration controlled by (a) erosion rate, (b) MAT, (c) soil regolith thickness and (d) catchment area. Dots are observations. The fitted lines show the model outputs of our final model M5 for different areal carbonate proportions. The other covariates are kept constant (MAT = 10°C, catchment area = 1000 km^2, soil regolith thickness = 15 m, erosion rate = 100 mm ka^{-1}).

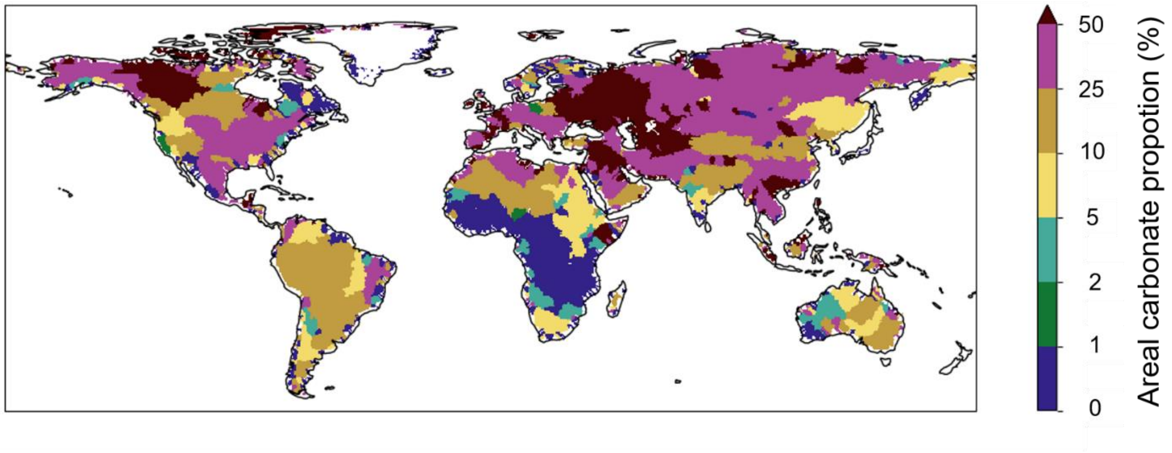

Supplementary Fig. 3: Global carbonate extent. The colors indicate the catchment average areal carbonate proportion of the world's major catchments. Areal carbonate proportion was calculated as the sum of the rock types "sc" and "sm" from the GLiM database[1]. The background map with the continent outlines is from naturalearthdata.com[2].

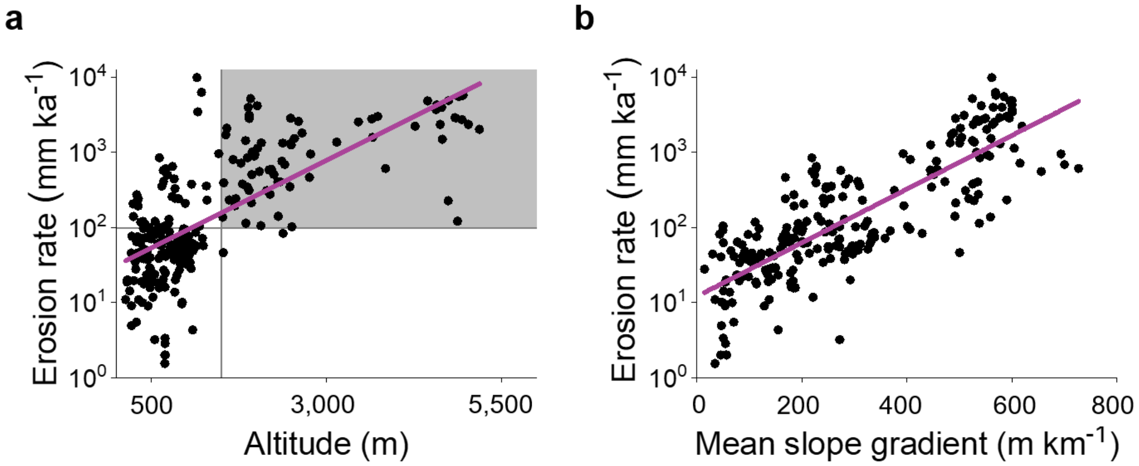

Supplementary Fig. 4: Erosion rate and topography. Erosion rate increases with both (**a**) altitude (R^2 = 0.5; p-value <2.2 x 10^{-16}) and (**b**) mean slope gradient (R^2 = 0.7; p-value <2.2 x 10^{-16}). (a) At mean altitudes >1500 m above mean sea level, catchments show intermediate to high erosion rate (grey-shaded box: erosion rate >100 mm ka^{-1}). (b) A high mean slope gradient promotes high erosion rates. The values for mean slope gradient were extracted from the OCTOPUS database[3]. Note: Although the reported R^2 refer to the linear regression of erosion rate and (a) altitude and (b) mean slope gradient, respectively, here the erosion rate is plotted on a logarithmic axis, indicating a non-linear relation.

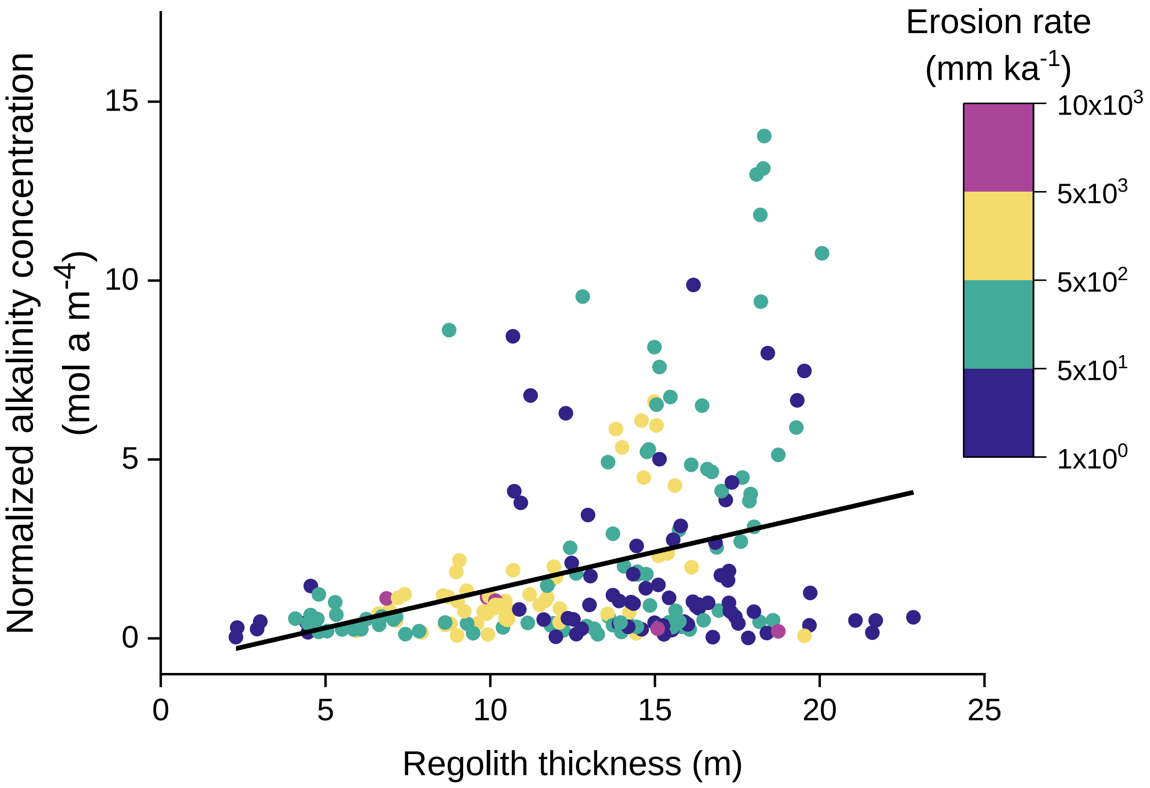

Supplementary Fig. 5: Normalized alkalinity concentration increases with soil regolith thickness. Normalized alkalinity concentration increases linearly with soil regolith thickness (R^2 = 0.1; p-value = 6.2 x 10^{-8}). Colors indicate erosion rate.

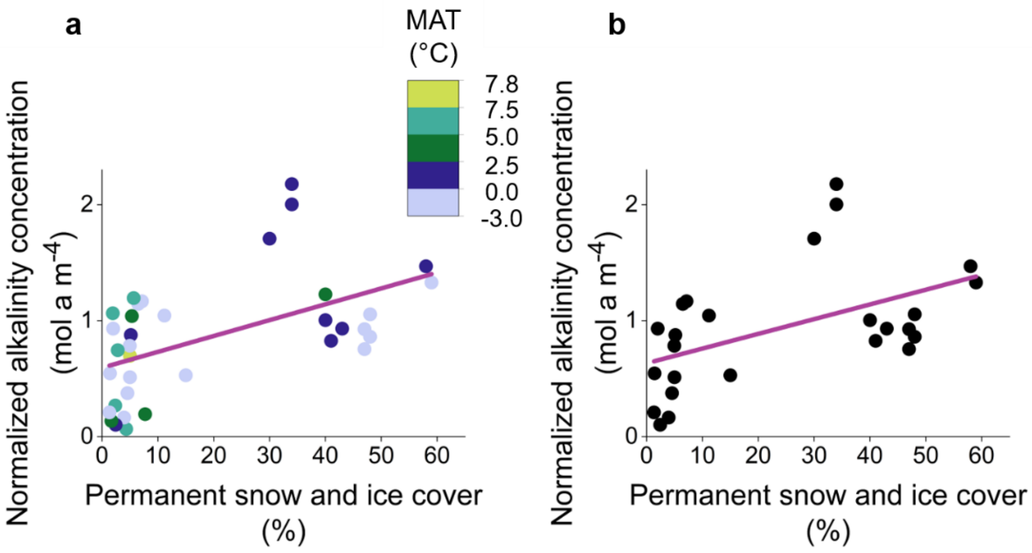

Supplementary Fig. 6: Normalized alkalinity concentration and permanent snow and ice cover.(**a**) Normalized alkalinity concentration increases linearly with permanent snow and ice cover for all catchments with permanent snow and ice cover >1% ($R^2 = 0.3$; p-value = 0.0011). Colors indicate mean annual temperature (MAT). (**b**) Normalized alkalinity concentration increases linearly with permanent snow and ice cover for all catchments with permanent snow and ice cover >1% and MAT <2.5 °C ($R^2 = 0.3$).

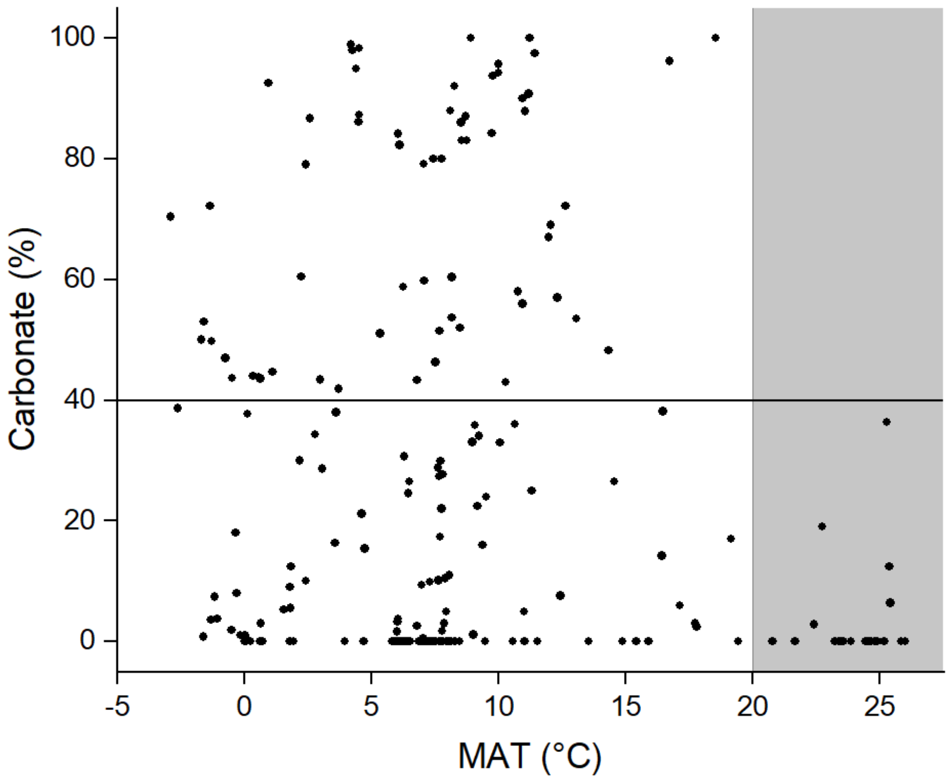

Supplementary Fig. 7: Areal carbonate proportion in relation to MAT. In catchments with a warm climate (grey-shaded box: MAT >20°C), the proportion of areal carbonate is generally low and does not exceed 40%.

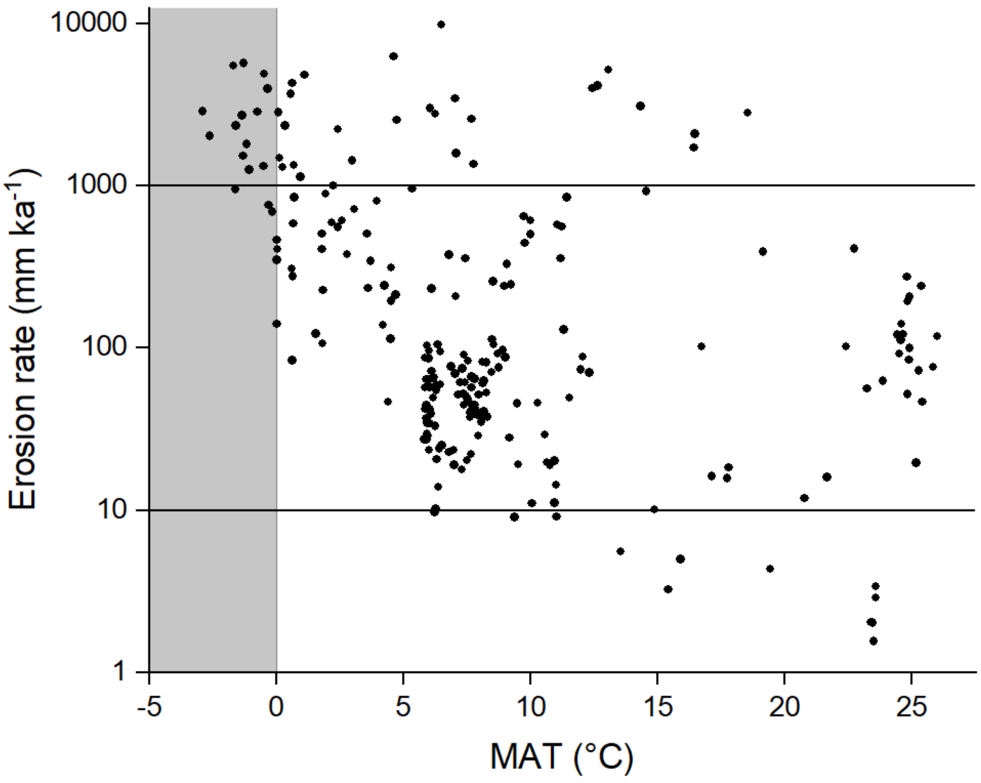

Supplementary Fig. 8: Correlation of erosion rate with MAT. Catchments with low MAT show only high erosion rates. Within the grey-shaded box (MAT < 0°C), erosion rates are very high, with almost all observations lying outside the *efficient erosion rate regime* (>1000 mm ka^{-1}).

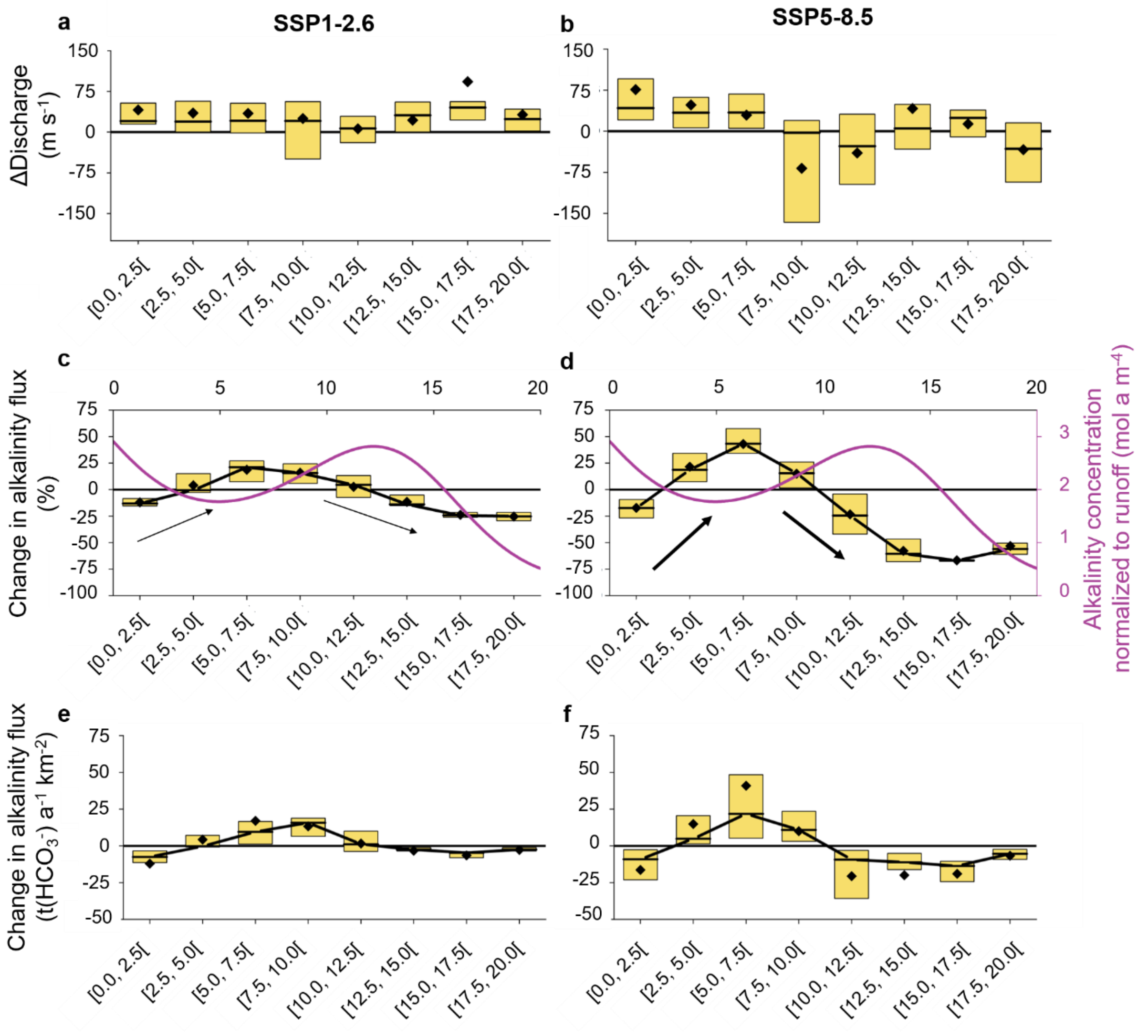

Supplementary Fig. 9: Alkalinity flux impacted by both changed alkalinity concentration and changed river discharge. The combined influence of alkalinity concentration and river discharge changes on the alkalinity flux is shown. In contrast, Fig. 3 shows the sole influence of the alkalinity concentration on the alkalinity flux (discharge is kept constant), since we cannot distinguish the individual influences of the two parameters within the combined signal. However, this cause attribution was the main focus of this paper. Simulated historical data (1980–2009) are contrasted with simulated future data affected by climate change according to (a, c, e) a low (SSP1-2.6) emissions scenario and (b, d, f) a high (SSP5-8.5) emissions scenario (2070–2099). (**a**, **b**) Difference in discharge (The difference in MAT can be seen in Fig. 3a and b.); (**c**, **d**) Relative change in alkalinity flux due to change in both MAT and discharge. The

schematic evolution of runoff normalized alkalinity concentration according to our model (from Fig. 2a) is shown for a better understanding. Thick arrows indicate that weathering responds more drastically to more rapidly changing temperature and discharge than to less rapidly changing temperature and discharge, indicated by the thin arrows; and (**e**, **f**) Absolute change in alkalinity flux due to change in both MAT and discharge. In contrast to Fig. 3, the discharge was not kept constant in this calculation, but instead the way it changes due to climate change was taken into account. For the calculation of the absolute alkalinity flux as specific mass flux, a molar mass of 61.02 g mol^{-1} for bicarbonate (HCO_3^-) was used, as at pH 7–9, the alkalinity concentration is approximately equal to the bicarbonate concentration[4,5]. Boxes indicate 0.25 and 0.75 quantiles and black diamonds show the arithmetic mean. Temperature projections are provided by the ISIMIP project[6] based on the GFDL-ESM4 data[7]. Discharge was simulated using the HydroPy global hydrology model[8].

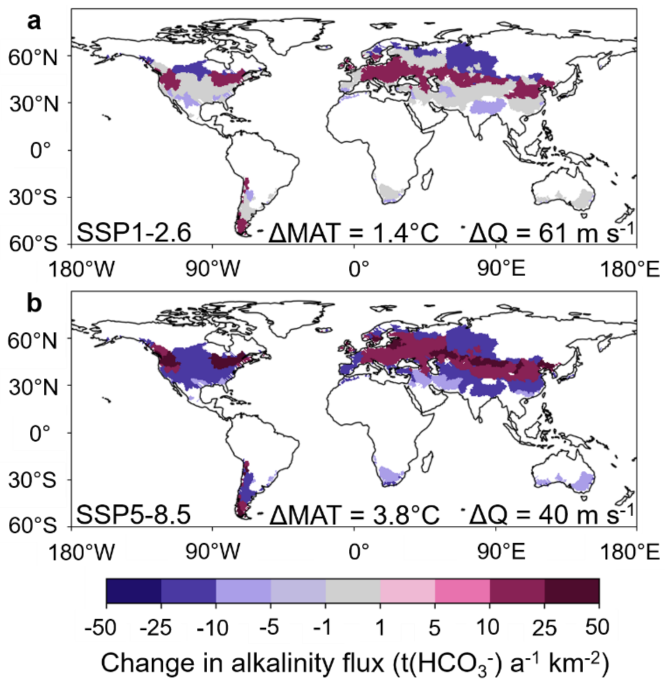

Supplementary Fig. 10: Projected change in alkalinity flux due to increased temperatures and changed river discharge. The combined influence of alkalinity concentration and river discharge changes on the alkalinity flux is shown. In contrast, Fig. 4 shows the sole influence of the alkalinity concentration on the alkalinity flux (discharge is kept constant), since we cannot distinguish the individual influences of the two parameters within the combined signal. However, this cause attribution was the main focus of this paper. Colors indicate the projected absolute change in alkalinity flux of catchments per temperature band for the historical temperature range of 0.0–20.0°C, globally, under scenarios (**a**) SSP1-2.6 and (**b**) SSP5-8.5. The mean change in MAT (ΔMAT) under SSP1-2.6 and SSP5-8.5 until the year of 2100 are projected to be 1.4 and 3.8°C, respectively. In contrast to Fig. 4, the discharge was not kept constant in this calculation, but instead the way it changes due to climate change was taken into account. The mean change in discharge (ΔQ) under SSP1-2.6 and SSP5-8.5 until the year of 2100 are projected to be 61 and 40 m s^{-1}, respectively. Catchment areas in white were excluded from the analysis, since their historical MATs were lower or higher than the temperature range of 0.0–20.0°C. For the calculation of the absolute alkalinity flux as specific mass flux, a molar mass of 61.02 g mol^{-1} for bicarbonate (HCO_3^-) was used, as at pH 7–9, the alkalinity

concentration is approximately equal to the bicarbonate concentration[4,5]. The background maps with the continent outlines in (a) and (b) are from naturalearthdata.com[2].

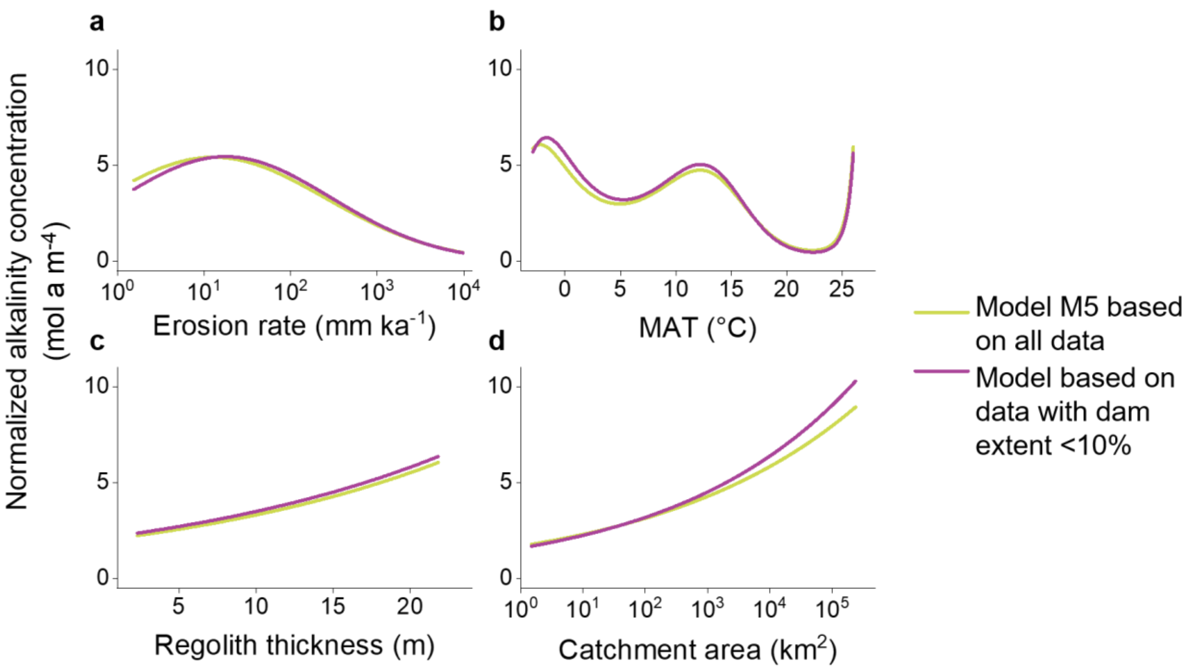

Supplementary Fig. 11: Influence of areal proportion affected by dams on alkalinity generation. The model function of M5 (based on complete training dataset, yellow line) is compared to a model function which is based on only the data with catchments that are characterized by an areal proportion affected by dams of <10% (purple line). Normalized alkalinity concentration as a function of (**a**) erosion rate, (**b**) MAT, (**c**) soil regolith thickness and (**d**) catchment area. The other covariates are kept constant (MAT = 10°C, catchment area = 1000 km^2, soil regolith thickness = 15 m, erosion rate = 100 mm ka^{-1}).

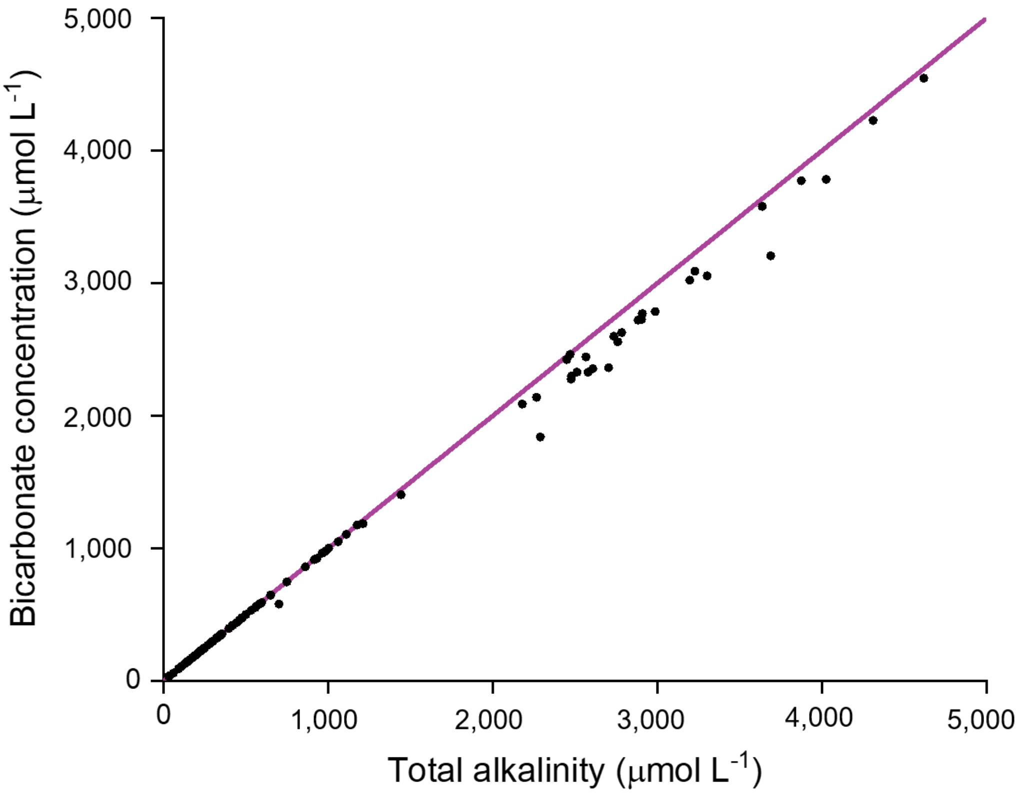

Supplementary Fig. 12: Bicarbonate concentration vs. total alkalinity concentration. Bicarbonate concentrations were calculated from total alkalinity and dissolved inorganic carbon for all samples taken during our sampling campaigns 2020. The data show almost equal proportions (1:1), with a greater variation at higher total alkalinity concentrations (>2000 µmol L^{-1}), which can be explained by an increase in the proportion of carbonate ion concentration.

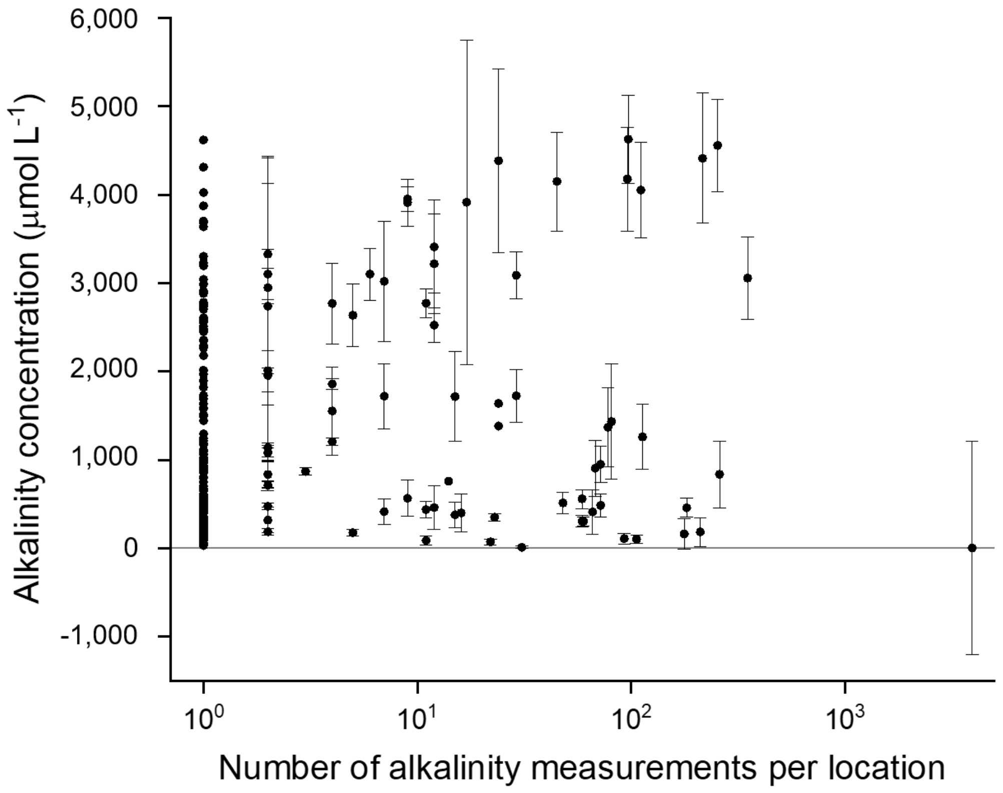

Supplementary Fig. 13: Alkalinity concentration, its standard deviation and the number of alkalinity measurements per location.

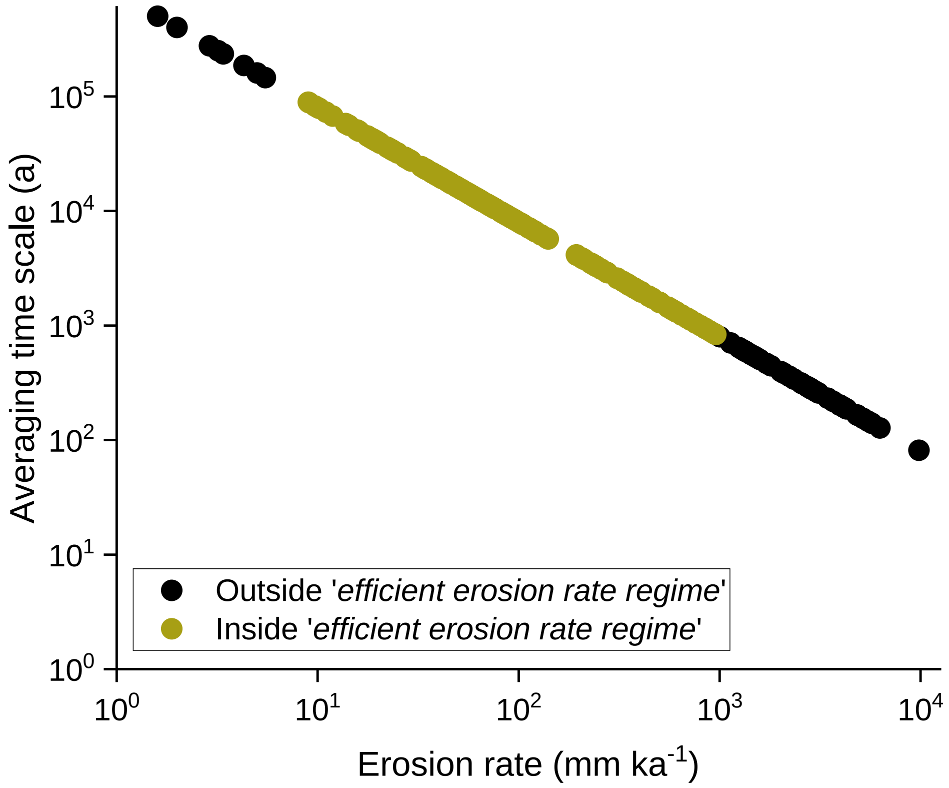

Supplementary Fig. 14: Time scales over which ^{10}Be-derived erosion rates integrate. All erosion rates from our data set are shown here. The absorption mean free path for fast nucleons in the upper meters of the Earth is ~155 g cm^{-2}. The density of regolith soil is on average ~1.95 g cm^{-3}. This results in an absorption depth of ~80 cm. Erosion rates within the limits of the *efficient erosion rate regime* (~10–1000 mm ka^{-1}), and the corresponding averaging times, are highlighted in gold.

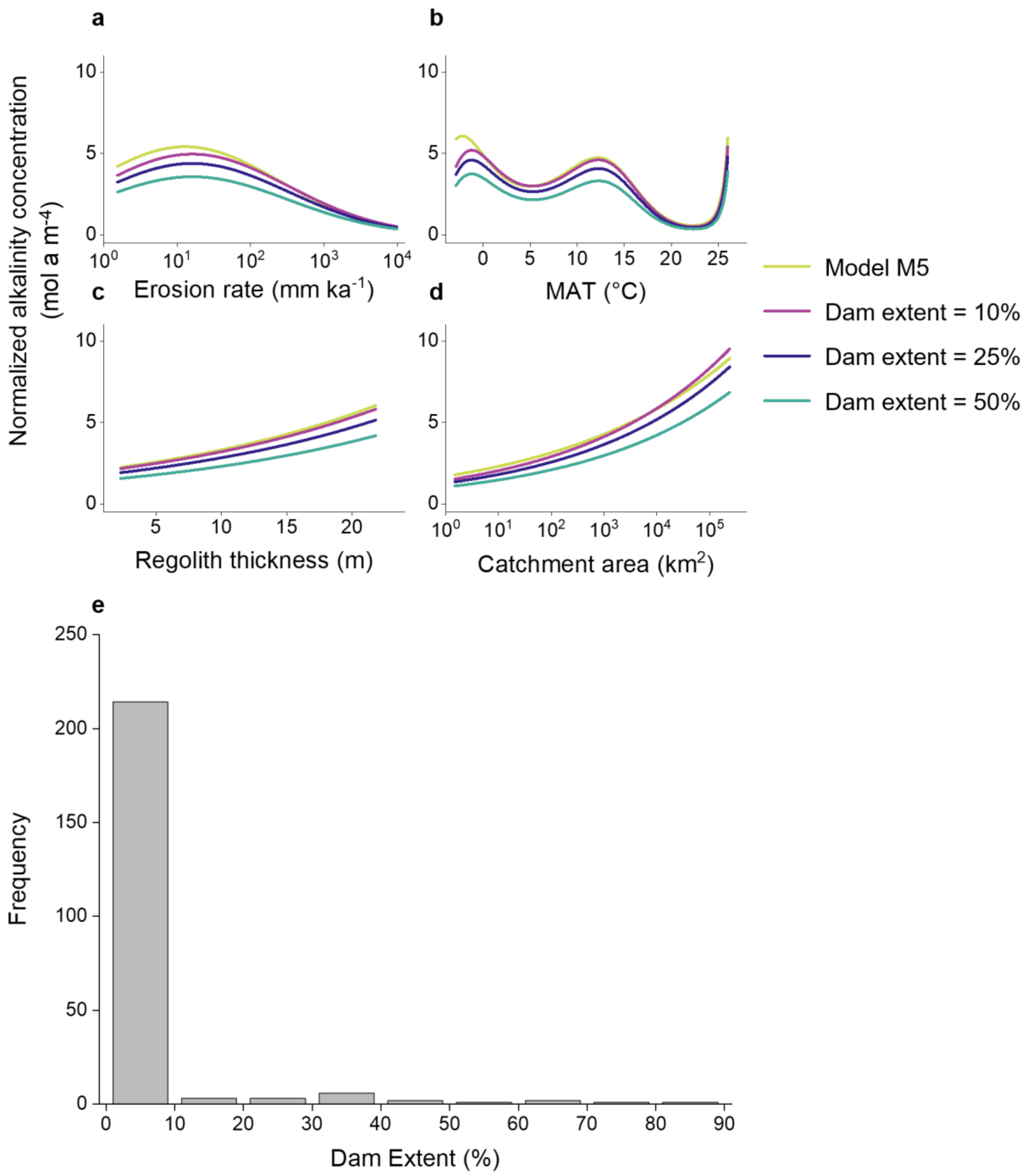

Supplementary Fig. 15: Comparison of model M5 with a GLM that includes dam extent as a covariate. The model function of M5 (yellow line) is compared to a GLM (generalized linear model) function that includes areal proportion affected by dams (i.e., dam extent) as a covariate in addition to the five covariates included in model M5. The purple, blue and green lines show the model outputs of that GLM for 10, 25 and 50% dam extent, respectively. Normalized alkalinity concentration as a function of (**a**) erosion rate, (**b**) MAT, (**c**) soil regolith thickness and (**d**) catchment area. The other covariates are kept constant (areal carbonate

proportion = 50%, MAT = 10°C, catchment area = 1000 km^2, soil regolith thickness = 15 m, erosion rate = 100 mm ka^{-1}). (**e**) Histogram showing frequencies of dam extent. Only a minor number of data points show a dam extent greater than 10%.

Supplementary Table 1. Comparison of model fit for normalized alkalinity concentration.

	Covariates	AIC	BIC	RSS	Ad. R^2	MSPE
**M1**	areal carbonate proportion	1024	1034	1077	0.342	4.78
**M2**	areal carbonate proportion<br>MAT (3rd degree pol.)	956	977	785	0.519	3.85
**M3**	areal carbonate proportion<br>MAT (3rd degree pol.)<br>ln(erosion rate) (2nd degree pol.)	868	895	527	0.680	2.85
**M4**	areal carbonate proportion<br>MAT (3rd degree pol.)<br>ln(erosion rate) (2nd degree pol.)<br>soil regolith thickness<br>ln(area)	794	829	378	0.763	1.89
**M5**	areal carbonate proportion<br>MAT (5th degree pol.)<br>ln(erosion rate) (2nd degree pol.)<br>soil regolith thickness<br>ln(area)	789	831	364	0.768	2.07
**GAM**	areal carbonate proportion<br>MAT<br>ln(erosion rate)<br>soil regolith thickness<br>ln(area)	709	824	214	0.849	2.52

All models (M1–M5) are generalized linear models (GLMs) with a natural logarithm as the link function. The model performance of a generalized additive model (GAM), also having a natural logarithm as the link function, but incorporating all covariates as smooth functions, is given as a reference. AIC: Akaike Information Criterion, BIC: Bayesian Information Criterion, RSS:

residual sum of squares, Ad. R^2: adjusted R^2, MSPE: mean squared prediction error, “pol.” = polynomial.

Supplementary Table 2. Comparison of model performance (RSS) for different temperature bands.

Temperature band (°C)	Number of observations	RSS (M3)	RSS (M4)	RSS (M5)
[-3,0)	16	11.3	3.1	1.4
[0,5)	45	206.6	136.7	124.8
[5,10)	106	1586.9	1342.9	1293.5
[10,15)	27	639.5	486.1	469.2
[15,20)	11	165.2	133.1	125.3
[20,27)	27	9.4	10.6	9.3

RSS: residual sum of squares.

Supplementary Table 3. Comparison of model performance (MSPE) for different temperature bands.

Temperature band (°C)	Number of observations	MSPE (M3)	MSPE (M4)	MSPE (M5)
[-3,0)	16	0.708	0.428	0.170
[0,5)	45	4.001	2.480	2.408
[5,10)	106	2.400	1.685	1.670
[10,15)	27	5.072	3.976	4.099
[15,20)	11	7.084	2.537	7.128
[20,27)	27	0.124	0.321	0.151

MSPE: mean squared prediction error.

References

1. Hartmann, J. & Moosdorf, N. The new global lithological map database GLiM. A representation of rock properties at the Earth surface. *Geochem. Geophys. Geosyst.* **13,** 119; 10.1029/2012GC004370 (2012).

2. Elson, P. *et al.* SciTools/cartopy: v0.21.1. *Zenodo*; 10.5281/zenodo.7430317 (2022).

3. Codilean, A. T. *et al.* OCTOPUS. An open cosmogenic isotope and luminescence database. *Earth Syst. Sci. Data* **10,** 2123–2139; 10.5194/essd-10-2123-2018 (2018).

4. Schroeder, E. D. Water Resources. In *Encyclopedia of physical science and technology*, edited by R. A. Meyers. 3rd ed. (Acad. Press, San Diego, 2002), pp. 721–751.

5. Dreybrodt, W. *Processes in Karst Systems. Physics, Chemistry, and Geology* (Springer, Berlin, Heidelberg, 1988).

6. Lange, S. & Büchner, M. *ISIMIP3b bias-adjusted atmospheric climate input data*. Available at https://doi.org/10.48364/ISIMIP.842396.1 (ISIMIP Repository, 2021).

7. Krasting, J. P. *et al. NOAA-GFDL GFDL-ESM4 model output prepared for CMIP6 CMIP* (Earth System Grid Federation, 2018).

8. Stacke, T. & Hagemann, S. HydroPy (v1.0). A new global hydrology model written in Python. *Geosci. Model Dev.* **14,** 7795–7816; 10.5194/gmd-14-7795-2021 (2021).
