# Supplementary material for: Alkalinity responses to climate warming destabilise the Earth’s thermostat
Source: Nat Commun. 2023 Mar 24;14:1648. doi: 10.1038/s41467-023-37165-w (PMC10039064; doi:10.1038/s41467-023-37165-w)
Supplement: Supplementary file 3 — Description of Additional Supplementary Files [file 41467_2023_37165_MOESM3_ESM.pdf]

Description of Additional Supplementary Files:

Supplementary Dataset 1: Source data for the analysis of the effect of climate change on alkalinity flux.

Source data for the analysis of the effect of climate warming from GFDL-ESM4 and HydroPy. We calculated the terrestrial alkalinity flux on a catchment basis for catchments with historical (1980-2009) MAT of 0.0- 20.0°C. Global temperature fields for historical and future (2070-2099) periods were provided by ISIMIP37 , based on scenario simulations conducted with the GFDL-ESM4 for the CMIP6 project68. Together with atmospheric fields from the same source (precipitation, radiation, humidity and wind), we were able to run the global hydrological model HydroPy43 to generate runoff and river discharge data. We compared historical with future data affected by climate warming according to a low (SSP1-2.6) and a high (SSP5-8.5) emissions scenario (SSP: shared socio-economic pathway). In contrast to MAT, we kept the other covariates in model M5 constant. Since no global map for 10Be erosion rates exists, we assumed an erosion rate of 100 mm ka-1 for all catchments. Further, we excluded all catchments whose values (in runoff, catchment area, soil regolith thickness) are not covered by our calibration data (i.e., runoff 239,000 km2 , soil regolith thickness 22.85 m). Even though the largest 105 catchments are not included in the calculations due to this data filter, we applied an extrapolation to the entire land surface area for the temperature range of 0.0-20.0°C (44,506,993 km2), because the most important controlling factors (areal carbonate proportion, MAT and erosion rate) were not affected. First, we fed our final model M5 with historical and future temperatures to yield estimates for historical and future normalized alkalinity, respectively. Second, we multiplied these with mean annual runoff (historical data: 1980-2009), which yielded alkalinity concentration. To assess only the effect of temperature change on alkalinity flux, we kept the discharge constant over time and multiplied both the historical and the future alkalinity concentration by historical mean annual discharge.
